# Amino-Functionalized Laponite Clay Material as a Sensor Modifier for the Electrochemical Detection of Quercetin

**DOI:** 10.3390/s22166173

**Published:** 2022-08-18

**Authors:** Delmas Vidal Tabe Ebunang, Kevin Yemele Tajeu, Chancellin Nkepdep Pecheu, Sherman Lesly Zambou Jiokeng, Arnaud Kamdem Tamo, Ingo Doench, Anayancy Osorio-Madrazo, Ignas Kenfack Tonle, Emmanuel Ngameni

**Affiliations:** 1 Electrochemistry and Chemistry of Materials, Department of Chemistry, University of Dschang, Dschang P.O. Box 67, Cameroon; 2 Institut für Anorganische Chemie und Strukturchemie, Heinrich-Heine-Universität Düsseldorf, 40204 Düsseldorf, Germany; 3 Laboratoire de Chimie Physique et Microbiologie pour les Matériaux et l’Environnement (LCPME), UMR 7564 CNRS—Université de Lorraine, 405, Rue de Vandœuvre, 54600 Villers-lès-Nancy, France; 4 Laboratory for Bioinspired Materials BMBT, Institute of Microsystems Engineering IMTEK-Sensors, University of Freiburg, 79110 Freiburg, Germany; 5 Freiburg Center for Interactive Materials and Bioinspired Technologies FIT, University of Freiburg, 79110 Freiburg, Germany; 6 Freiburg Materials Research Center FMF, University of Freiburg, 79104 Freiburg, Germany; 7 Laboratory of Analytical Chemistry, Department of Chemistry, University of Yaounde 1, Yaoundé P.O. Box 812, Cameroon

**Keywords:** amino-laponite, quercetin, modified electrode, voltammetry

## Abstract

In this work, an electrode modified with an amino-functionalized clay mineral was used for the electrochemical analysis and quantification of quercetin (QCT). The resulting amine laponite (LaNH_2_) was used as modifier for a glassy carbon electrode (GCE). The organic–inorganic hybrid material was structurally characterized using X-ray diffraction, Fourier transformed infrared spectroscopy (FTIR), scanning electron microscopy (SEM), thermogravimetric analysis (TGA) and CHN elemental analysis. The covalent grafting of the organosilane to the clay backbone was confirmed. The charge on the aminated laponite, both without and with the protonation of NH_2_ groups, was evaluated via cyclic voltammetry. On the protonated amine (LaNH_3_^+^)-modified GCE, the cyclic voltammograms for QCT showed two oxidation peaks and one reduction peak in the range of −0.2 V to 1.2 V in a phosphate buffer–ethanol mixture at pH 3. By using the differential pulse voltammetry (DPV), the modification showed an increase in the electrode performance and a strong pH dependence. The experimental conditions were optimized, with the results showing that the peak current intensity of the DPV increased linearly with the QCT concentration in the range from 2 × 10^−7^ M to 2 × 10^−6^ M, leading to a detection limit of 2.63 × 10^−8^ M (S/N 3). The sensor selectivity was also evaluated in the presence of interfering species. Finally, the proposed aminated organoclay-modified electrode was successfully applied for the detection of QCT in human urine. The accuracy of the results achieved with the sensor was evaluated by comparing the results obtained using UV–visible spectrometry.

## 1. Introduction

**Scheme 1:**
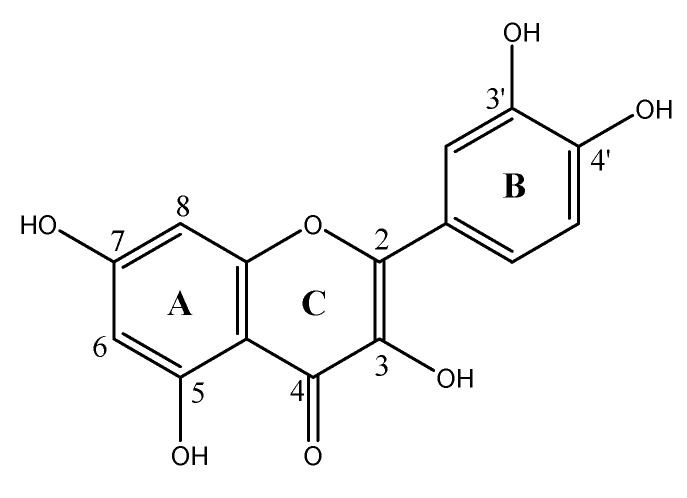
Chemical structure of the quercetin (QCT) molecule.

Flavonoids include a wide range of natural polyphenolic compounds belonging to the group of phytonutrients found in all fruits and vegetables [1]. After their isolation and identification in 1936 by Szent-Gyorgyi [2], several studies have shown their strong antioxidant capacity linked to their ability to bind free radicals in the cellular matrix [3,4]. In addition to this, flavonoids can also exhibit pro-oxidant activity [5]. Although the daily consumption by humans is estimated at around hundred milligrams [6], studies have indicated that a high consumption rate of flavonoids reduces cardiovascular diseases [4,6]. Flavonoids are also anti-inflammatory, antibacterial, anticancer and anti-ischemic agents [4,6]. For commercial purposes, plants rich in antioxidant compounds enhance the properties of the foods with which they are associated [7]. One of these molecules is quercetin (3, 3′, 4′, 5, 7-pentahydroxy- flavone, QCT, Scheme 1). It differs from others of the same class, such as iso-quercetin and rutin, by the presence of a hydroxyl instead of a glycoside group in the C ring, which gives it some specific properties [8]. In the literature, there are several works highlighting the medicinal properties of QCT [9,10].

Found in many vegetables and fruits, QCT is also a constituent of many common pharmaceuticals and dietary supplements [11]. It has received considerable attention due to its beneficial effects on human health. Quercetin can be detected and quantified in many matrices using spectrophotometric [12,13], chromatographic [14] and coulometric methods [15]. Although these methods are highly efficient and sensitive, the analysis times are quite long. Electrochemical methods appear as an alternative for the fast detection and oxidation of QCT in aqueous solution [16]. Due to their low cost and simplicity, electrochemical methods offer the possibility to build portable devices for the detection and quantification of flavonoids. These devices work with modified electrodes. Several studies have highlighted the electrochemical analysis of QCT on different modified electrodes, including a multiwalled carbon nanotube (MWCNT) paste electrode [16,17], a glassy carbon electrode (GCE) modified with carbon nanotubes (CNTs) dispersed in nafion [18], a GCE modified with microbial succinoglycan monomers and multi-walled carbon nanotubes [19], and a novel carbon paste electrode chemically modified with purified Arabic gum for the direct quantification of both QCT and rutin in different natural fruits and human urine [20]. Although these materials are effective, they can often present a certain degree of impurities, making their application in electroanalysis complex. It is, therefore, necessary to develop new electrode materials with high purity.

Synthetic clay minerals appear to be promising electrode materials. They have properties that are very beneficial for catalysis and absorption [21]. However, in their natural state their properties are weak, but the possibility for structural modifications that they allow is an asset. Organosilanes are widely used as modifiers for increased sensitivity and selectivity [22,23]. Studies on the surface modification of natural clays, silica and amorphous alumina have been reported in the literature [24,25]. Laponite is a synthetic clay mineral consisting mainly of layered hydrated magnesium silicates. It is obtained hydrothermally and is widely used in industrial and consumer products. Although its structure and composition are like that of its natural counterpart hectorite, its specific surface area is 350 m²/g and its crystalline disk-shaped particles are 1.0 nm thick, with an average radius of 20 nm and a charge of about 0.73 (meq/g) [26]. The surface modification of laponite has allowed: (i) the preparation of organosilasequioxane–laponite clay films on solid electrodes and their use as (bio)electrochemical sensors [27]; (ii) the immobilization of several enzymes in clay matrices [28]; (iii) the immobilization of catalysts [29]; (iv) the removal of unwanted biomolecules from extractors and suspension [30]. The literature reports some studies on the use of natural clays such as kaolinite and halloysite for the detection and adsorption of quercetin [31,32]. To our knowledge, the use of amino-functionalized laponite (synthetic clay) for the detection and quantification of quercetin (QCT) in aqueous solution has not yet been studied.

The main goal of this study was to test an amino-functionalized laponite as a modifier of a glassy carbon electrode (LaNH_3_^+^/GCE) in electrochemical sensor applications for the quantitative analysis of QCT. Specifically, this work was focused firstly on the evaluation of the ionic exchange capacity of the sensor and secondly on the ability to electroanalyze QCT. Cyclic voltammetry (CV) was used to study the electrochemical behavior of the QCT with the developed organic- and inorganic-functionalized sensor. Then, differential pulse voltammetry (DPV) was used to build the calibration curve for the electroanalytical quantification of QCT flavonoids in human urine samples.

## 2. Materials and Methods

### 2.1. Laponite Clay Mineral and Chemicals

The laponite clay mineral with the structural formula Na_0.7_(Si_8_Mg_5.5_Li_0.3_)O_20_(HO)_4_ [30,33] originated from the Source Clays Repository of the Clay Minerals Society, Purdue University (West Lafayette, IN, USA). It was used without further purification. All reagents were of analytical grade. The quercetin (98%) (QCT) was purchased from Sigma-Aldrich. The (3-(2-aminoethylamino)propyl)trimethoxysilane (AEPTMS, Sigma-Aldrich, Taufkirchen, Germany) was used as a grafting agent. The caffeine, D(+) glucose, ascorbic acid, tartaric acid, catechol, paracetamol, uric acid, ciprofloxacin and sodium sulfite were obtained from Sigma-Aldrich; citric acid monohydrate from J.T. Baker; and potassium hexacyanoferrate (III and II) from Prolabo. When required, the pH of the prepared solutions was adjusted either with HCl or NaOH solutions.

### 2.2. Organofunctionalization of Laponite Material

The functionalization process for the laponite was adapted from a procedure reported in a previous study [8]. A known amount of laponite (2 g) was dispersed in 15 mL of toluene and mixed under a N_2_ flow for 10 min. Then, to the mixture maintained under stirring, 4 mL of (3-(2-aminoethylamino)propyl)trimethoxysilane (AEPTMS) was added dropwise. The whole was refluxed for 3 h under stirring. The resulting modified laponite was filtered and washed several times using toluene to remove the unreacted silane residue. The final product was dried at 100 °C overnight, gently ground in a mortar and stored for further characterization and use. The organofunctionalized laponite is hereafter referred to as LaNH_2_.

### 2.3. Physicochemical Characterizations of Amino-Functionalized and Unmodified Laponites

The physicochemical characterization of laponite before and after its chemical modification was performed by X-ray diffraction (XRD), Fourier transform infrared (FTIR) spectroscopy, elemental analysis, thermal analysis and scanning electron microscopy (SEM).

The XRD was performed on a STOE Stadi-p diffractometer (STOE and Cie GmbH, Darmstadt, Germany) operating at 40 kV and 30 mA, with Cu Kα1 radiation (40 kV, 30 mA and λCu = 1.54056 Å).

The FTIR spectra were acquired using a Bruker α-PFTIR spectrophotometer (Bruker, Germany) within a wavenumber range of 500–4000 cm^−1^, with a resolution of 4 cm^−1^. Here, 200 scans were collected for each spectrum.

Elemental analysis was performed with a Euro Vector CHNSO element analyzer (Euro EA 3000) or a vario MICRO Cube (HEKAtech GmbH, Wegberg, Germany).

The thermal decomposition of the samples was investigated via thermogravimetric analysis (TGA, NETZSCH). Approximately 10 mg of each dried sample was weighed in an aluminum pan and heated from 50 to 1000 °C under nitrogen atmosphere with a heating rate of 10 K/min

The SEM images were taken on Amray 1610 Turbo instrument (Anray Inc., Bedford, MA, USA) at an accelerating voltage of 15 kV. Each sample was deposited on a conductive tab pressed onto a specimen holder and coated with gold under vacuum using a sputter coater.

### 2.4. Electroanalytical Analyses and Preparation of the Glassy Carbon Working Electrode

Each glassy carbon electrode (Ø = 3 mm) was polished with alumina pastes of different particle sizes (5, 1 and 0.5 µm). It was then placed in a 1:1 ethanol–water solution and sonicated for 10 min to remove traces of alumina. The thin-film clay-modified glassy carbon working electrodes were obtained by drop coating 5 μL of either a laponite clay, LaNH_2_ or acidified LaNH_2_ (LaNH_3_^+^) dispersion (5 g/L) on the active surface of the glassy carbon electrode (GCE), followed by drying at 100 °C in an oven for 5 min. The LaNH_3_^+^ suspension was obtained by dispersing 5 mg of LaNH_2_ in 1 mL of 0.1 M hydrochloric acid. The prepared working electrodes were denoted La/GCE, LaNH_2_/GCE and LaNH_3_^+^/GCE for the GCE coated with raw laponite, LaNH_2_ and acidified LaNH_2_, respectively.

The charge transport ability and mass transfer of each of the modified GCEs was evaluated via cyclic voltammetry in 0.01 M HCl containing an (Fe(CN)_6_)^3−^ redox probe, by comparing the signals obtained of the La/GCE, LaNH_2_/GCE and LaNH_3_^+^/GCE. The oxidation–reduction process for QCT was also investigated via cyclic voltammetry, in a 50:50 (v/v) mixture of a 0.1 M PBS and ethanol solution set at pH 3 and containing 5 × 10^−4^ M of QCT. Differential pulse voltammetry was applied for sensing studies, using the following parameters: pulse amplitude 50 mV; step potential, 5 mV; equilibrium time, 5 s. A mixture (v/v) of decimolar phosphate-buffered solution (PBS) and 95% ethanol was used as a supporting electrolyte. All experiments were performed at room temperature. The urine samples chosen as real samples were obtained from a healthy human volunteer in the laboratory. The recovery experiments in human urine were performed as follows. A given volume of the urine sample was diluted in the blank-supporting electrolyte placed in the electrochemical cell. A known amount of QCT was then added. Each recovery rate was calculated by comparing the results obtained before and after the addition of the analyte, using DPV as described above.

The electrochemical manipulations were performed using an OrigaStat Potentiostat–Galvanostat (OrigaLys ElectroChem SAS, Rillieux-la-Pape, Rhône, France) controlled with the OrigaMaster 5 software, whose cell consists of three electrodes: a reference electrode (Ag/AgCl/KCl 3 M, Metrohm), a stainless-steel counter electrode and a glassy carbon working electrode. A GENESYS 10S UV–visible spectrophotometer was used to collect the UV–visible spectra of the QCT at a maximum absorbance of 373 nm.

## 3. Results

### 3.1. Structural Characterization of Laponite and Amino-Functionalized Laponite Electrode Modifier

#### 3.1.1. X-ray Diffraction and Infrared Spectroscopy Analyses

**Figure 1:**
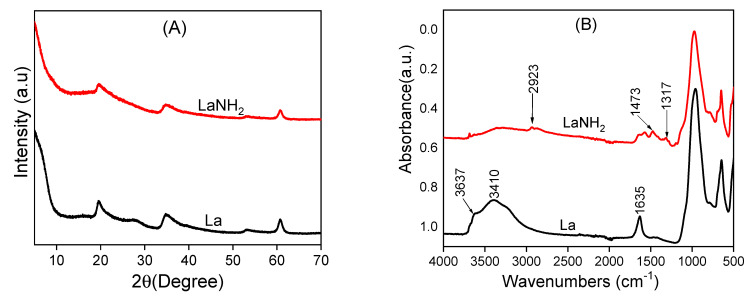
X-ray diffraction (XRD) patterns (**A**) and infrared spectra (**B**) of pristine laponite La and amino-functionalized laponite LaNH_2_ materials.

The X-ray diffraction (XRD) patterns of laponite and its amino-functionalized counterpart showed diffraction peaks that corresponded to the primary diffractions of the clay material, corresponding to the peaks of laponite in four planes (Figure 1). By comparing the laponite XRD patterns before and after the surface functionalization with AEPTMS, it can be seen that there was no change since the same peaks were obtained. This reveals that during the grafting of AEPTMS, the crystalline structure and crystallinity of the laponite are preserved. The IR spectra of the La and LaNH_2_ materials are compared in Figure 1.

Two main characteristics can be observed, indicating that the starting material undergoes a significant change during grafting with AEPTMS:

(i) The decrease in the intensity of the OH stretching bands at 3637 cm^−1^ attributed to the internal Al- or Mg-linked OH group of the clay after grafting with the organosilane molecules, suggests the participation of hydroxyl groups [34,35,36,37] in the interactions of AEPTMS with the clay surface. In addition, the decrease in intensity of some remarkable vibration bands observed at 3410, 1635, 973 and 647 cm^−1^, respectively, due to the physisorbed water, the OH vibrations and the Si-O and Si-O-Si stretching can be explained by the prolonged warming during the functionalization of the clay;
(ii) The appearance of new bands in the modified clay spectrum, which are not present in the unmodified clay spectrum, centered at 2923, 1473 and 1317 cm^−1^ are attributed to C-H deformation bonding, asymmetric stretching vibrations and C-H and C-H bands of amino-functionalized laponite (LaNH_2_) [38,39,40,41]. Characteristics (i) and (ii) provide strong evidence for the presence of organic fragments in the organic film, consistent with previous reports [42].

#### 3.1.2. Thermal Analyses

**Figure 2:**
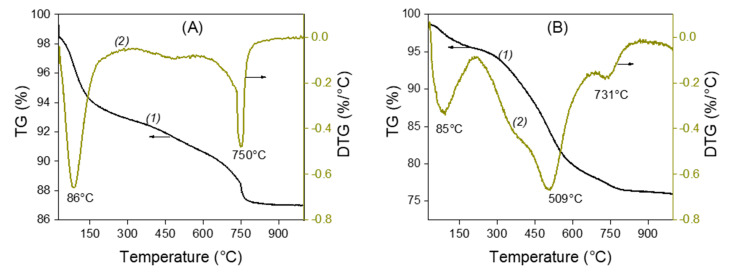
Thermogravimetric analysis (curves 1) and differential thermal gravimetry (curves 2) curves of unmodified La (**A**) and amino-functionalized laponite LaNH_2_ (**B**).

Figure 2 shows the thermogravimetric analysis (TGA) and differential thermal gravimetric (DTG) curves for unmodified (La) and amino-functionalized (LaNH_2_) laponite clays. In Figure 2A, which corresponds to raw laponite, two mass loss decays can be observed: the first one at 86 °C related to the evaporation of the water adsorbed in the clay, and the second one at 750 °C due to the dehydroxylation of the clay layers; that is, their collapse under the effect of heating. In Figure 2B, which corresponds to the LaNH_2_ characterization, three mass loss characteristic temperatures can be observed. The first one at 85 °C, with a loss mass of 7%, is due to the adsorbed water. The second one at about 509 °C, which was absent on the thermogram of the raw laponite (La), is related to the destruction of the AEPTMS modification moieties, whose melting point is exactly 509 °C, which confirms the interaction between the AEPTMS and the laponite. The presence of organic modifiers has the effect of lowering the dehydroxylation temperature of the layers to 731 °C compared to 750 °C in unmodified clay. The same results were observed by Tajeu et al. for the thiol-grafted laponite [33].

#### 3.1.3. Scanning Electron Microscopy

**Figure 3:**
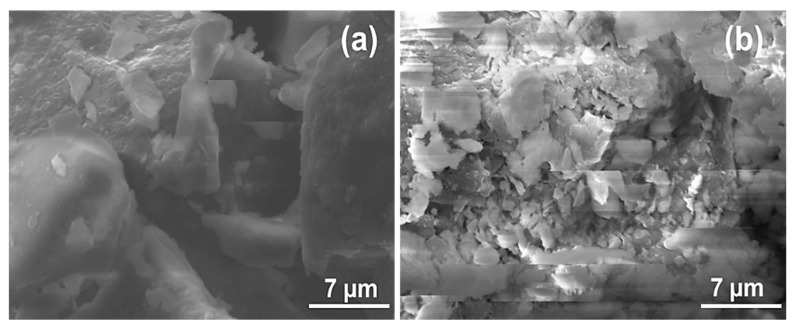
Scanning electronic microscopy images of unmodified La (**a**) and amino-functionalized laponite LaNH_2_ (**b**).

The analysis of the SEM micrographs of La and LaNH_2_ (Figure 3) showed a well-defined morphology, characterized by non-homogeneous assembled clay particles (Figure 3a). As shown in Figure 3a, the laponite particles present a platelet-like morphology. However, a tendency towards aggregation can be observed, leading to agglomerates with a regular shape and to a compact material (Figure 3a). In the case of the LaNH_2_ material (Figure 3b), the particles are apparently smaller and consist of aggregates of fine homogeneous and disordered sheet particles. The modification apparently caused homogeneous disorder and a reduction in particle size. It can be concluded that after the functionalization, the organosilane fragments were aggregated on the surface of the laponite.

### 3.2. Evaluation of the Ionic Exchange Capacity of Protonated Amino-Functionalized Laponite (LaNH_3_^+^)

**Figure 4:**
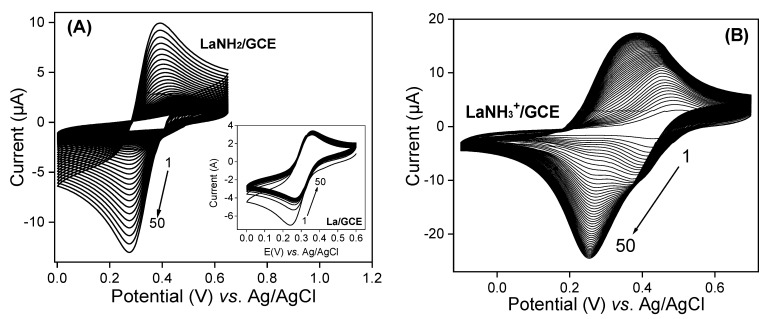
Multisweep cyclic voltammograms recorded in 10^−2^ M HCl containing 5 × 10^−4^ M (Fe(CN)_6_)^3−^ on a glass carbon electrode (GCE) modified with (**A**) LaNH_2_ and (**B**) LaNH_3_^+^ materials. The inset in Figure 4A represents the same experiment on the GCE covered by a thin film of unmodified La.

The chemical modification of laponite via the grafting of AEPTMS is expected to change its surface reactivity. In this regard, the ionic exchange properties of LaNH_2_ and LaNH_3_^+^ were investigated using multisweep cyclic voltammetry in the presence of (Fe(CN)_6_)^3−^ ions used as a standard redox probe. Figure 4A shows the voltammograms recorded in HCl at pH 2 containing 5 × 10^−4^ M of (Fe(CN)_6_)^3−^ on the GCE modified with a thin film of LaNH_2_, while the inset presents the signal obtained with pristine laponite. The latter is characterized by a well-defined reversible voltammogram resulting from the physical absorption of the anions on the surface of the laponite. As the number of cycles increases, a decrease in peak intensity can be observed. This is due to the repulsion between the negatively charged surface and anions. By replacing La/GCE with LaNH_2_/GCE under the previous conditions, one can observe on Figure 4A that the peak current increases gradually with continuous cycling.

This is due to the gradual protonation of –NH_2_ groups on the La surface, which gradually attract negatively charged ions from the solution. To confirm that the absorption of anions is governed by electrostatic attraction, LaNH_2_ was acidified to LaNH_3_^+^, and in the same conditions the uptake of the redox probe was more pronounced as the peak current at saturation was more important (~18 µA) compared to LaNH_2_/GCE (10 µA), as shown in Figure 4B. Indeed, recent studies have demonstrated the strong affinity displayed by amine functions towards compounds carrying negative charges of an electrostatic nature [41,43,44,45]. This affinity allows the immobilization or easy absorption of negatively charged species in the amino and protonated matrices when electrostatic interactions are involved [46,47,48,49,50,51,52]. By comparing the results obtained on LaNH_2_/GCE and LaNH_3_^+^/GCE, a large difference can be noted. On the multicyclic voltamograms of (Fe(CN)_6_)^3−^ recorded on LaNH_2_/GCE, a reduction peak at ~0.25 V can be observed. On the other hand, on LaNH_3_^+^/GCE, two reduction peaks appear: the first one at ~0.4 V, which disappears slowly, and a second one at ~0.25 V, stabilizing with the number of scans. The observation of such behaviour related to the coupled (Fe(CN)_6_)^3−/4−^ on our LaNH_3_^+^ material is not common. As Tonle and co-workers reported in 2004 [53], one might suggest that the signals at ~0.4 V are due to “free” (Fe(CN)_6_)^3−^ species in the solution phase that were exchanged for the electrolyte anions prior to their electrochemical reduction, whereas the voltammetric signals at ~0.25 V could be attributed to (Fe(CN)_6_)^3−^ species bound to LaNH_3_^+^. This hypothesis is also supported by the fact that electron hopping in clay films dominates at low to moderate charges, while physical scattering limitations become prominent at high concentrations of the electroactive probe in the material [54]. These data support the successful grafting of AEPTMS on the surface of the La clay material.

### 3.3. Electrochemical Behavior of QCT on LaNH_3_^+^/GCE

**Figure 5:**
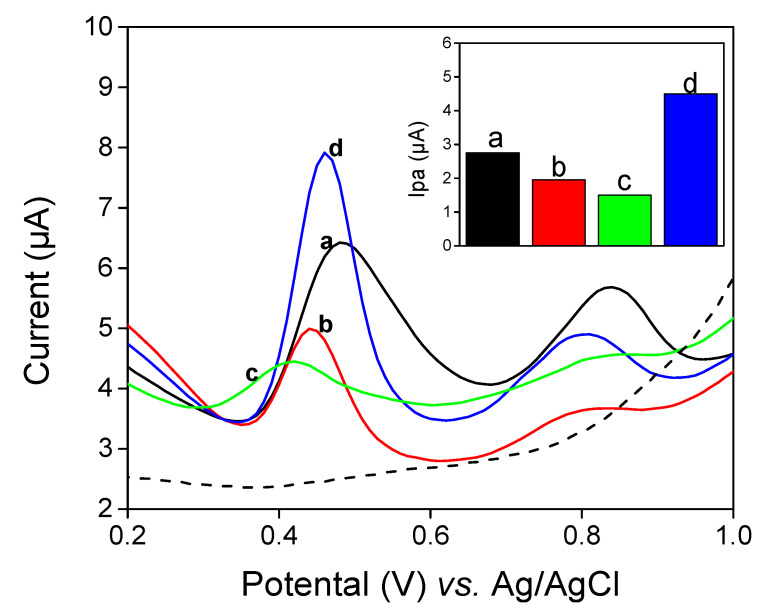
Differential pulse voltammograms (DPV) for 10^−4^ M of quercetin (QCT) in (v/v) phosphate buffer and EtOH mixture solution (pH 2) in (**a**) LaNH_2_/GCE, (**b**) La/GCE, (**c**) GCE and (**d**) LaNH_3_^+^/GCE working electrodes.

Figure 5 shows the different pulse voltammograms of 0.1 × 10^−3^ M QCT in 50:50 (v/v) phosphate buffer and EtOH solutions at (pH 2) on (Figure 5a) LaNH_2_/GCE, (Figure 5b) La /GCE, (Figure 5c) bare GCE and (Figure 5d) LaNH_3_^+^/GCE. On these electrodes, QCT exhibited two oxidation peaks. However, the peak current obtained on LaNH_3_^+^/GCE is greater than that recorded on LaNH_2_/GCE, which is 2-fold less important than the peak current of La/GCE. This last one is almost reduced compared to the unmodified GCE. This is attributed to the low electrical conductivity of La. As expected, AEPTMS is supposed to improve the sensitivity of the laponite clay. This sensitivity increases when AEPTMS is acidified. Furthermore, it has an electrocatalytic effect on the oxidation of QCT, since it causes the potential shift toward the low value (from +0.47 V to +0.42 V). It was, therefore, used to develop a sensitive method of determining QCT.

### 3.4. Effect of Scan Rate

**Figure 6:**
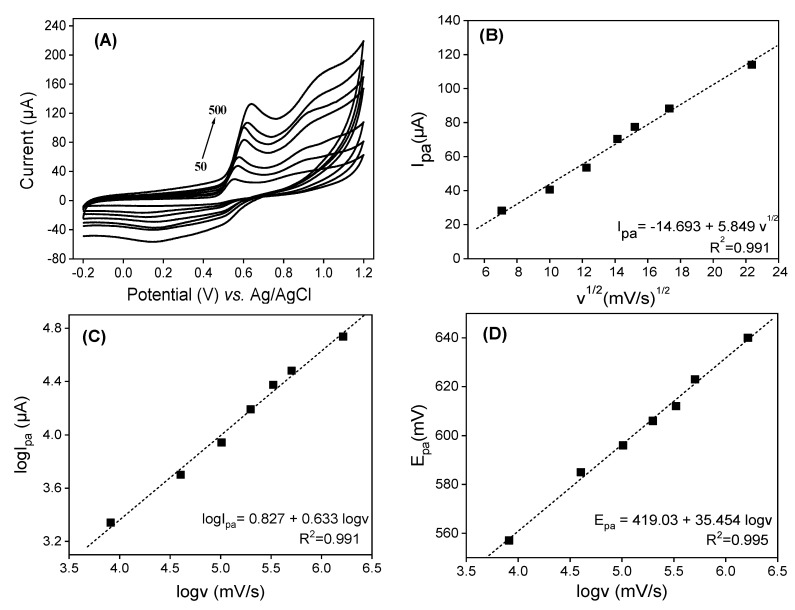
(**A**) Variations in scan rate on LaNH_3_^+^/GCE immersed in 0.1 M of 50:50 (v/v) phosphate buffer and EtOH mixture solution (pH = 2) in the presence of 5 × 10^−4^ M of QCT. (**B**) A plot of I_pa_ = f (v^1/2^). (**C**) A plot of logI_pa_ = f (logv). (**D**) A plot of E_pa_ = f (logv).

In order to elucidate the electrode reaction of QCT, the effects of the potential scan rate (v) and peak potential (Epa) on the peak currents (ipa) were studied using cyclic voltammetry. Figure 6 depicts cyclic voltammograms obtained in (v/v) phosphate buffer and EtOH (pH = 2) containing 0.5 × 10^−3^ M of QCT on LaNH_3_^+^/GCE over the scan rate (v) range of 50 to 500 mV.s^−1^. As shown in Figure 6A, an increase in peak currents (i_p_) with v is noted with a shift of peak potentials to positive values. The plot of i_pa_ = f (v^1/2^) in Figure 6B shows the linear increase in the anodic peak current with the square root of the scan rates (v^1/2^). The linear regression equation i_pa_ (mA) = −14.69 + 5.85 v^1/2^ (mV.s^−1^)^½^ (R^2^ = 0.991) close to 1 indicates a diffusion-controlled reaction. By plotting log (i_pa_) = f (logv) (Figure 6C), a good linear relationship is obtained with the equation log i_pa_ (mA) = 0.827 + 0.633 log v (mV.s^−1^), with R^2^ = 0.991. A slope of 0.633 obtained, greater than 0.5, which besides the diffusion, indicates the presence of a weak adsorption process of the electroactive species on the electrode.

The good linear relation obtained between the peak potential and logv (Figure 6D), described by the equation E_pa_ (mV) = 419.03 + 35.45 Logv (mV.s^−1^) with R^2^ = 0.995, combined with the electrochemical theory of Laviron [49] for an irreversible process, allowed us to calculate kinetic parameter values such as the electron transfer coefficient α, the number of transfer electrons n and the standard heterogeneous reaction rate constant k. In order to study the adsorption-controlled irreversible process at the electrode, Laviron [55] suggested the following equation:

(1) $$E={\;E}^{0}+\left(\frac{2.303\mathrm{RT}}{\alpha \mathrm{nF}}\right)\log\left(\frac{{\mathrm{RTK}}^{0}}{\alpha \mathrm{nF}}\right)+\left(\frac{2.303\mathrm{RT}}{\alpha \mathrm{nF}}\right)\mathrm{logv}$$

In Equation (1), E^0^ represents the formal redox potential, R is the universal gas constant (8.314 J mol K^−1^), T is the room temperature (298.15 K), α is the electron transfer coefficient, n is the number of transferred electrons, F is the Faraday constant (96,480 C.mol^−1^), K^0^ is the standard rate constant of the surface reaction and v is the scan rate. By plotting the I_Pa_ vs. Log(v) curve (Figure 6C), using the slope the value αn = 1.625 was determined, and with Bard and Faulkner’s equation [56] *,* Equation (2) α was calculated:

(2) $$\alpha \left(mV\right)=\frac{47.7}{E_{pa}-E_{pa}/2}\;$$

Considering E_pa_/2 is the potential measured at half of the peak current value, α = 0.738 was obtained, allowing us to evaluate the number of electrons exchanged to 2.20 ≈ 2. By plotting the graph of E_pa_ = f(v) and extending it to the ordinate access (v = 0) [51], a value of 0.567 V was obtained for E^0^, providing the standard rate constant of the surface reaction K^0^ (1.071 s^−1^).

### 3.5. Effect of Supporting pH on the Quercetin Response

**Figure 7:**
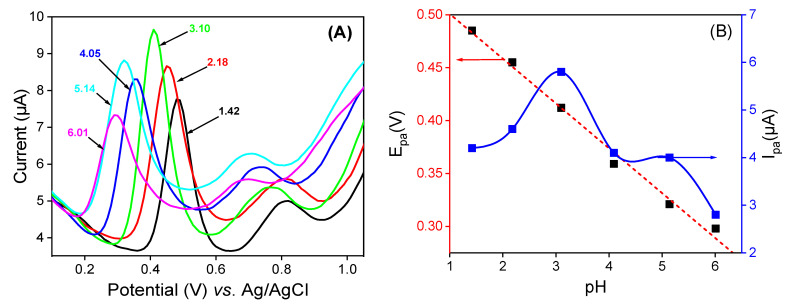
(**A**) Variation in pH values of (v/v) phosphate buffer and EtOH mixture solution on LaNH_3_^+^/GCE in the presence of 10^−4^ M of QCT. (**B**) The curves of i_pa_ = f (pH) and Epa = f(pH).

**Scheme 2:**
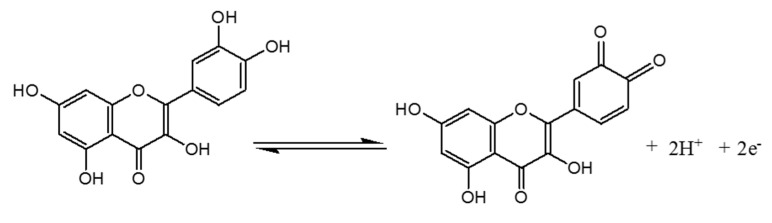
The proposed electrochemical redox reaction of quercetin.

The pH of the supporting electrolyte medium is one of the most important parameters that can strongly affect the electrode reaction, mostly when the latter involves protons. DPV studies were conducted in 0.1 M of 50:50 (v/v) phosphate buffer and EtOH at different pH values containing 10^−4^ M of QCT. The voltammograms shown in Figure 7A were obtained. As can be seen, the oxidation peak potential (E_pa_) of QCT shifts to negative values with the increase in pH, indicating that the oxidation reaction involves protons [8]. The plot of the current height against the pH (Figure 7B) showed the best signal and maximum current at pH = 3, which was chosen as the optimum pH for the electro-oxidation of QCT. As shown in Figure 7B, a good linear relationship was noted between E_pa_ and pH, with the equation E_pa_ (V) = −0.042 pH + 0.543 (R^2^ = 0.991). Scheme 2 below highlights the proposed electrochemical redox reaction of quercetin involving two electrons and two protons. A slope value of 0.0423V/pH units was obtained, which was approximately equal to the theoretical value of the Nernst slope of 0.059 V/pH at 25 °C [57], indicating that the number of electrons involved in the electro-oxidation of QCT is equal to the number of protons.

### 3.6. DPV Calibration Curve and Detection Limit

**Figure 8:**
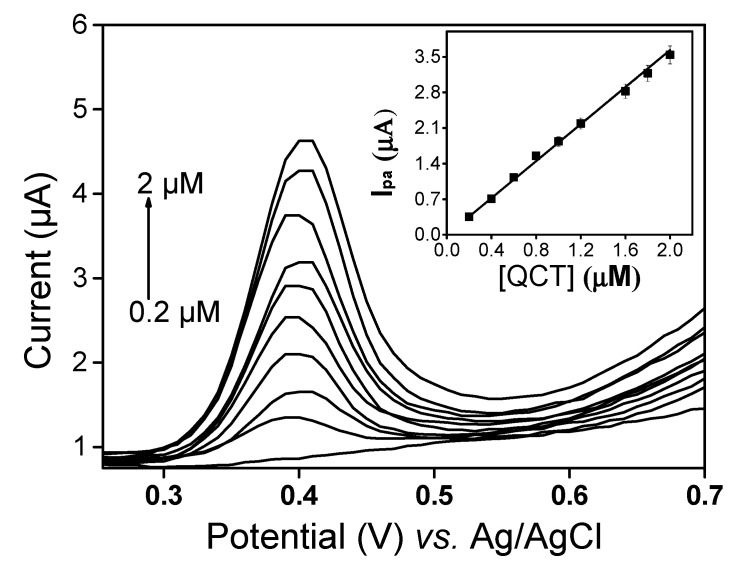
DPV voltammograms of QCT in a LaNH_3_^+^/GCE sensor at different concentrations (0.0, 0.2, 0.4, 0.6, 0.8, 1.0, 1.2, 1.6, 1.8, 2.0 µM). Inset: Plot of i_pa_ vs. quercetin concentrations (QCT) in a 50:50 (v/v) phosphate buffer and EtOH mixture solution (pH 3).

Under optimal conditions, the detection performance of LaNH_3_^+^/GCE was investigated in the quantification of QCT. Figure 8 shows the voltammograms of QCT obtained from DPV in the range between 2 × 10^−7^ and 2 × 10^−6^ M. In this range, a peak current was observed to increase with the concentration and calibration curve (inset Figure 8), and with the equation ipa (µA) = −0.007 + 1.81 [QCT] (µM) is obtained.

The correlation coefficient of 0.997 indicated a good linearity between the peak current and QCT concentration on LaNH_3_^+^/GCE. The limit of detection calculated using the formula 3s_D_/m, where s_D_ is the standard deviation and m the slope of the calibration curve of current against concentration, was 0.0263 × 10^−6^ M for QCT. The results obtained demonstrate that LaNH_3_^+^/GCE has good analytical performance and can be used to determine QCT in solution. A comparison of the proposed electrode with the recently reported methods listed in Table 1 showed that LaNH_3_^+^/GCE is superior to some existing electrodes.

### 3.7. Repeatability, Reproducibility and Stability of the Electrode

The repeatability of the LaNH_3_^+^/GCE sensor was evaluated for five repetitive measurements of differential pulse voltammetry in the presence of 100 × 10^−6^ M QCT. The relative standard deviation (% RSD) was estimated at 3.1%, showing the good repeatability of the method used. The reproducibility of the sensor was studied on ten different electrodes and a relative standard deviation (% RSD) of 0.52% was obtained. The stability of the LaNH_3_^+^ film on the electrode surface was evaluated after five successive measurements on the same electrode for a recovery rate of 96.04%.

### 3.8. Interference Analysis

**Figure 9:**
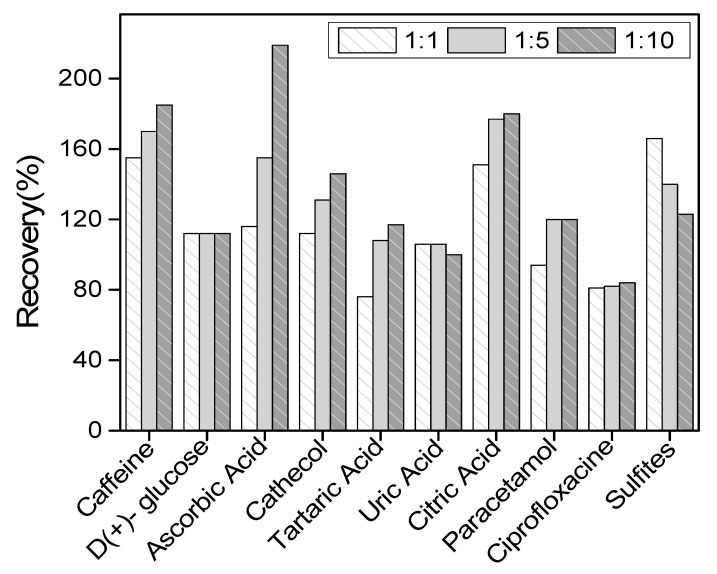
Influence of various potentially interfering compounds on the differential pulse voltammetry (DPV) response in the presence of quercetin (QCT) at 10^−4^ M.

The selectivity of LaNH_3_^+^/GCE towards QCT on the current response was investigated in the presence of some organic molecules that are likely to coexist with QCT in real medium. In the preconcentration medium containing a fixed amount of QCT ((QCT) = 10^−4^ M), each suspected interference species was added at a concentration of a 1-, 5- or 10-fold excess of QCT. The ten interfering species selected were ascorbic acid, tartaric acid, uric-acid, citric acid, D-(+)-glucose, paracetamol, ciprofloxacin, caffeine, sulfites ions and catechol. The obtained result indicated the percentage increase in the peak current of QCT, as shown in Figure 9 below. From this figure, it can be observed that the percentage increases with the addition of tartaric acid, catechol, caffeine, paracetamol and ascorbic acid, with this last one being the most interfering molecule. This increase in peak current could be explained by the superposition of signals, as the oxidation potentials of QCT and those of some of the interferents were close to each other. Tartaric acid is the least interfering molecule. A decreasing peak current was observed with uric acid and sulfites, probably due to competition reactivity at the electrode surface. No interference was observed with D-(+)-glucose and ciprofloxacin (constant current).

### 3.9. Detection of QCT in Urine Samples

Our LaNH_3_^+^/GCE sensor was applied to the detection of QCT in a urine sample and UV–visible spectrophotometry was used for comparative purposes. In UV–vis spectroscopy, and like most flavones and flavonols, quercetin has two major absorption maxima, one in the range of 240–285 nm (band II belonging to the benzoyl system of ring A) and the other in the range 300–400 nm (band I belonging to the cinnamoyl system of ring B) [61]. The urine sample was diluted 100-fold in a phosphate buffer–ethanol mixture (50:50, v/v) before analysis. Our sample was then spiked with increasing concentrations (0.4 × 10^−6^, 0.8 × 10^−6^, 1.2 × 10^−6^ and 1.6 × 10^−6^ M) of QCT and analyzed using the sensor under previously established optimal conditions and with UV–visible spectrophotometry. The recovery rates obtained and presented in Table 2 show that whatever the method used, the amount of QCT detected increases with the amount added. The recovery rates (above 95%) obtained with the sensor show that it can be very useful and efficient for the electroanalysis of QCT in urine samples. However, using UV–visible spectrophotometry, the recovery rates above 105% highlight the complexity in terms of the organic molecules of the analyzed medium, which is very sensitive to ultraviolet–visible.

## 4. Conclusions

In this work, we developed a glassy carbon electrode modified with an amino- functionalized laponite (LaNH_3_^+^) film, which was then applied to the selective detection of QCT in an aqueous solution and in a urine sample. The kinetic parameters governing the electrochemical oxidation reaction of QCT at the surface of our electrode were determined. In addition to this, the detection conditions were optimized, allowing us to establish a detection limit of 0.0263 × 10^−6^ M over a concentration range of 0.2 × 10^−6^ to 2 × 10^−6^ M. The sensor was then successfully applied in a real environment. This work demonstrates the potential of synthetic clay minerals as electrode materials for the detection and quantification of trace molecules in biological media.

## Figures and Tables

**Table 1:** Comparison of the performances of the LaNH_3_^+^/GCE sensor with those of other GCE-modified electrodes applied to the electroanalysis of quercetin.

GCE Modifier	Linear Range (×10^−6^ M)	Detection Limit (×10^−6^ M)	Real Sample	References
Graphene	0.006–10	0.0036	Apple, onion	[57]
MWCNTs dispersed in polyacrylic acjd	2–11	0.2	Onion	[35]
poly (safranine O)	0.01–8	0.005	Wine, Fruit Juice	[58]
TEOS@Fe_2_O_3_	1–9	0.092	Apple tea	[59]
PtNPs/PEDOT-MeOH	0.04–91	0.052	Apple juice	[60]
Aminated laponite	0.2–2	0.0263	Human urine	This work

MWCNTs: multiwalled carbon nanotubes; TEOS@Fe_2_O_3_: tetraethylorthosilicate coated to magnetic Fe_2_O_3_; PtNPs: Pt nanoparticles; PEDOT: poly(hydroxymethylated-3,4-ethylenedioxythiophene).

**Table 2:** Determination of QCT in urine sample.

		DPV		UV-Visible	
Sample	QCT Added (×10^−6^ M)	QCT Found (× 10^−6^ M)	Recovery (%)	QCT Found (×10^−6^ M)	Recovery (%)
1	0.4	0.38	96.20	0.55	137
2	0.8	0.77	97.70	0.94	117
3	1.2	1.17	97.20	1.39	116
4	1.6	1.56	97.50	1.73	108

## Data Availability

Not applicable.
